# HER2 testing in gastric cancer: results of a German expert meeting

**DOI:** 10.1007/s00432-017-2374-x

**Published:** 2017-03-11

**Authors:** Florian Lordick, Salah-Eddin Al-Batran, Manfred Dietel, Timo Gaiser, Ralf-Dieter Hofheinz, Thomas Kirchner, Hans H. Kreipe, Sylvie Lorenzen, Markus Möhler, Alexander Quaas, Christoph Röcken, Josef Rüschoff, Andrea Tannapfel, Peter Thuss-Patience, Gustavo Baretton

**Affiliations:** 1 Universitätsklinikum Leipzig, Universitäres Krebszentrum (UCCL), Liebigstraße 20, 04103 Leipzig, Germany; 2 UCT University Cancer Center Frankfurt, Frankfurt am Main, Germany; 3 Institut für Pathologie, Charité Universitätsmedizin Berlin Campus Mitte, Berlin, Germany; 4 Pathologisches Institut der Universitätsmedizin Mannheim, Mannheim, Germany; 5 Universitätsmedizin Mannheim, Mannheim, Germany; 6 Pathologisches Institut der Ludwig-Maximilians-Universität München, Munich, Germany; 7 Institut für Pathologie, Medizinische Hochschule Hannover, Hanover, Germany; 8 Medizinische Klinik des Klinikums rechts der Isar, Munich, Germany; 9 Medizinische Klinik und Poliklinik, Universitätsmedizin Mainz, Mainz, Germany; 10 Institut für Pathologie, Uniklinik Köln, Cologne, Germany; 11 Institut für Pathologie Christian-Albrechts-Universität, Kiel, Germany; 12 Institut für Pathologie Nordhessen u. Targos GmbH, Kassel, Germany; 13 Georgius Agricola Stiftung Ruhr, Institut für Pathologie der Ruhr-Universität Bochum am Berufsgenossenschaftlichen Universitätsklinikum, Bochum, Germany; 14 Charité-Universitätsmedizin Berlin, Campus Virchow-Klinikum, Berlin, Germany; 15 Institut für Pathologie, Universitätsklinikum Carl Gustav Carus, Dresden, Germany

**Keywords:** HER2 testing, Gastric cancer, Consensus, Quality assurance, Trastuzumab

## Abstract

Valid HER2 testing is essential for optimal therapy of patients with HER2-positive gastric cancer and the correct use of first-line chemotherapy. While testing for HER2 status in breast cancer is routinely performed, this is not the case for HER2 testing in gastric cancer and it is usually only performed on clinician request. An interdisciplinary German expert group (pathologists and clinicians) took the challenges of HER2 testing in gastric cancer as an opportunity to address essential aspects and questions for the practical use of HER2 testing in this indication. The recommendations made in this manuscript reflect the consensus of all participants and reflect their opinions and long-term experience in this field.

## Introduction

Valid human epidermal growth factor receptor 2 (HER2) testing is essential for the optimal care of patients with advanced gastric cancer and the correct use of first-line drug therapy. Prerequisites for the accurate diagnosis of HER2 status are the availability and quality of samples, quality-assured histopathological diagnosis and close communication between pathologists and clinicians in the interpretation of the findings with regard to their therapeutic consequences. While today all breast cancers are routinely tested for the HER2 status, this is not yet the case for HER2 testing in gastric cancer and it is often only performed when requested by the clinician. One underlying reason for this is due to the fact that trastuzumab is only approved for the treatment of patients with advanced or metastatic gastric cancer, while the breast cancer label also includes patients with early HER2-positive breast cancer (Summary of Product Characteristics Herceptin® i.v. 2015). Therefore, HER2 testing in gastric cancer only becomes relevant for therapeutic decision-making at the advanced or metastatic stage. Another aspect is that the smaller number of specimens tested means that experience of HER2 testing in gastric cancer is not as extensive as in breast cancer. It is hoped that through the establishment of gastrointestinal oncology centers and clinics, where patient care is more disease-oriented and focused, testing of the HER2 status will become the standard for any patient with gastric cancer as recommended in guidelines. A group of German experts took the challenges of HER2 testing in gastric cancer as an opportunity to address essential issues for the practical application of HER2 testing in this indication from the perspective of pathologists and clinicians.

## Which gastric cancers should be tested for HER2?

### Consensus

It is advocated that all gastric carcinomas which are diagnosed in advanced stages are immediately tested for HER2 expression. In principle, it would be desirable that all cases of adenocarcinoma of the stomach and the gastroesophageal junction should be tested for HER2 expression in parallel to the initial histo-diagnostic procedure (so-called “up-front testing”), however, the cost implications and therapeutic relevance of this approach are controversial.

### Background/rationale

**Fig. 1:**
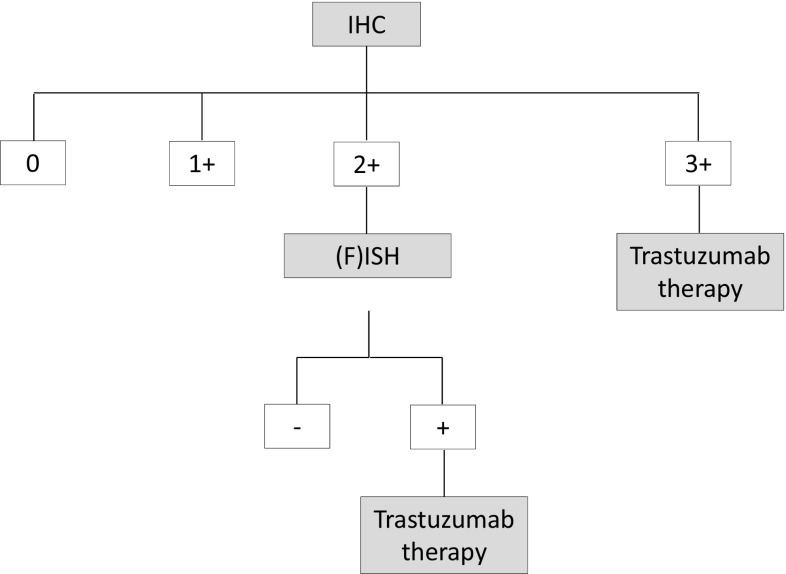
HER2-testing algorithm in adenocarcinoma of the stomach and gastroesophageal junction (modified acc. to Rüschoff et al. 2010)

In the vast majority of cases, gastric cancer is diagnosed in an advanced, metastatic or inoperable stage and is a rapidly progressive disease (Horner et al. 2009; Kamangar et al. 2006). HER2 overexpression is found in approximately 7–34% of gastric cancers, depending on location and histological subtype, and is a predictive marker for response to trastuzumab, which is currently the only approved HER2-targeted therapeutic option for the first line treatment of patients with metastatic gastric cancer (Bang et al. 2010; Brien et al. 1998; Chung et al. 2009; Gravalos and Jimeno 2008; Park et al. 2006; Rüschoff et al. 2012; Takehana et al. 2002; Tanner et al. 2005). The use of trastuzumab is indicated for metastatic gastric cancer with HER2 overexpression (defined as immunohistochemistry [IHC] staining pattern 3 +, or IHC2 + and in-situ hybridization [ISH] +) based on a proven survival benefit (Bang et al. 2010; Moehler et al. 2011). Therefore, according to the German S3 guideline, HER2 status should be determined prior to palliative tumor therapy with trastuzumab (Moehler et al. 2011). According to the current recommended testing algorithm, an IHC-based assessment is the initial test for HER2 overexpression. In inconclusive IHC cases (2 +) *HER2* gene amplification status for further clarification with ISH testing is required (see Fig. 1). Various ISH techniques are available using different labeled probes including fluorescence-ISH (FISH), silver-ISH (SISH) and chromogen-ISH (CISH). While FISH has been the most commonly used technique, other ISH procedures have become accepted more recently. When selecting an ISH method, S/CISH are reported to be slightly superior to FISH as they allow evaluation of the results in the context of the tissue structure (Shipley 2002).

Trastuzumab in combination with capecitabine or 5-fluorouracil and cisplatin is approved and indicated for the treatment of adult patients with HER2-positive metastatic adenocarcinoma of the stomach or gastroesophageal junction who have not received prior anti-cancer treatment for their metastatic disease. Approval was based on the phase III Trastuzumab for Gastric Cancer (ToGA) study, which showed a significant survival benefit for patients treated with trastuzumab plus chemotherapy (capecitabine or 5-fluorouracil and cisplatin) as compared with chemotherapy alone (Summary of Product Characteristics Herceptin^®^ i.v. 2015; Bang et al. 2010). The beneficial effects were mainly detected in patients with the highest level of HER2 protein overexpression, defined by a 3 + score by IHC, or a 2 + score by IHC and a positive FISH result (Summary of Product Characteristics Herceptin^®^ i.v. 2015; Bang et al. 2010).

The scoring system shown in Table 1 is recommended to evaluate IHC staining patterns in gastric cancer (Rüschoff et al. 2010). The test to detect *HER2* gene amplification status is considered positive if the ratio of the *HER2* gene copy number per tumor cell to the chromosome 17 copy number is greater than or equal to 2 (*HER2*/CEP17-Ratio ≥2) (Summary of Product Characteristics Herceptin^®^ i.v. 2015). In gastric cancer, the *HER2*/CEP17 ratio is decisive; in cases with higher gene counts (≥6.0) and negative ratio (<2.0) the tumor should be considered as HER2-positive in accordance with the updated recommendation for breast cancer (Wolff et al. 2013).

**Table 1:** Modified HER2 immunoscoring for gastric cancer (Rüschoff et al. 2010) (modified acc. to Hofmann et al. 2008)

Staining intensity: IHC-Score	Surgical specimen staining pattern	Biopsy specimen staining pattern	HER2 status
0	No reactivity or membranous reactivity in <10% of tumor cells	No reactivity or membranous reactivity in any (or <5) tumor cell(s)	*Negative*
1+	Very weak membranous reactivity in ≥10% of tumor cells	Tumor cell cluster with a very weak membranous reactivity irrespective of percentage of tumor cells stained (at least 5 tumor cells)	*Negative*
2+	Weak to moderate complete, basolateral or lateral only membranous reactivity in at least 10% of tumor cells	Tumor cell cluster with a weak to moderate complete, basolateral or only lateral membranous reactivity irrespective of percentage of tumor cells stained (at least 5 tumor cells)	*Equivocal* (ISH assessment required)
3+	Strong complete, basolateral or lateral only membranous reactivity in at least 10% of tumor cells	Tumor cell cluster with a strong complete, basolateral or lateral only membranous reactivity irrespective of percentage of tumor cells stained (at least 5 tumor cells)	*Positive*

Currently, trastuzumab should only be used in patients with metastatic gastric cancer (MGC) whose tumors have HER2 overexpression as defined by IHC2 + and a confirmatory positive ISH result, or by an IHC 3 + result. To ensure accurate and reproducible results, validated and standardized assay methods should be used (Summary of Product Characteristics Herceptin^®^ i.v. 2015). Therefore, HER2 testing should be performed in a specialized laboratory, which can ensure validation of the testing procedures and which is staffed by trained personnel (Summary of Product Characteristics Herceptin^®^ i.v. 2015).

To give patients access to optimal therapy, the experts endorse immediate, rapid and quality-assured histopathological testing for HER2 status as the basis for decisions regarding drug treatment for metastatic gastric cancer. HER2 testing can be performed on endoscopic biopsies or on surgical specimens. Early knowledge of HER2 status is crucial not only for the use of a targeted therapy, but also for the selection of the optimal chemotherapy regimen. If chemotherapy is started before the HER2 status is known, a change of the first initiated chemotherapy regime may be required at a later date.

Taking into consideration cost implications and therapeutic relevance, the following recommendations for early testing of HER2 status were made:

- HER2 testing should ideally be performed as so-called “up-front testing” (i.e. at initial diagnosis) in all cases of adenocarcinoma of the stomach or gastroesophageal junction. Should this not be warranted, HER2 testing can also be performed upon request of the clinician who submitted the sample for analysis (see Table 2). Outside of clinical trials, first line use of trastuzumab in line with its approval status is currently only a consideration for patients with HER2 positive metastatic gastric cancer.
- When histopathology is requested on a potentially relevant tissue sample, the pathologist should advise the requesting clinician about the option to perform up-front HER2 testing (i.e. at any initial diagnosis of gastric cancer).
- When up-front testing is performed, the pathologist should initially carry out an IHC-based assessment. In inconclusive cases (IHC2+), *HER2* amplification status should be assessed with ISH according to the currently recommended test algorithm.

**Table 2:** HER2-testing in gastric cancer—What does the pathologist want to know from the clinician?

Submission of at least 5 tumor-containing biopsies collected from different tumor sites to allow for valid testing
When requesting the report, active communication as to whether HER2 testing is required
Full clinical data (precise description of the sampling point, patient history, initial diagnosis, relapse, treatment etc.)

## Sample quality and quantity, and their impact on HER2 testing

The minimum number of biopsies and tumor cells required for robust HER2 testing was discussed.

### Consensus

Five tumor-containing biopsies from different areas of the tumor should be aimed for. If the number of biopsies for reliable HER2 testing is insufficient and/or in inconclusive cases (e.g. IHC2+, not analyzable or borderline ISH result), a rebiopsy is required. This also applies where sample quality does not allow valid assessment of HER2 status. In inconclusive cases, other tumor-containing tissue should also be assessed, e.g. metastatic tissue in addition to the primary tumor, if applicable. For validation of IHC2+ cases with ISH at least 50 tumor cells should be available. If further drug therapy is required in patients with HER2-negative primary tumors, additional tumor samples should be tested to check current tumor status; metastatic tissue is also suitable for this purpose.

### Background/rationale

According to the German S3-guideline, when malignancy of the stomach or gastroesophageal junction is suspected, a minimum of eight biopsies should be taken from all suspicious lesions to ensure a reliable diagnosis (Moehler et al. 2011). From the pathologist’s perspective, for reliable diagnosis of HER2 status the specific number of biopsies is less relevant than the quality of biopsies and the number of tumor-containing biopsies obtained. Due to the high intratumoral heterogeneity of gastric carcinoma and the focal nature of HER2 staining in up to 30% of cases (Bang et al. 2010; Rüschoff et al. 2010, 2012; Warneke et al. 2013), the experts recommend taking biopsies from different areas of the tumor to ensure that representative material is obtained sufficiently and to avoid false negative results.

A rebiopsy is recommended in the following cases:

In patients with a HER2-negative primary tumor, a rebiopsy should be performed in cases of recurrence. If possible, this should be done prior to the treatment decision ensuring a potentially HER2-positive tumor is not overlooked. In a recent study, the chance of HER2-positivity on rebiopsies in initially HER2-negative gastric cancer was 8.7% for the primary site and 5.7% for metastases. In particular, the chance of HER2-positivity on rebiopsy was relatively high in initially IHC2+/ISH-findings at the primary site (25%) and in liver metastases (17.2%) (Park et al. 2016).

If biopsy material is insufficient or its quality is low, the pathologist should give clear feedback to the sender that the results of HER2 testing may not be robust. Based on their extensive experience, the participants of the expert meeting concluded that *at least five-tumor-containing biopsies* should be aimed for (Gullo et al. 2015; Tominaga et al. 2016) (see Table 2). If less than five tumor-containing biopsies are available, there is an increased risk of a false negative result. If a second gastroscopy is not possible, the HER2 status can also be assessed in other tumor-containing material (metastasis or surgical specimens).

At least 50 tumor cells should be available for the evaluation of ISH in case of inconclusive IHC findings. The rationale for this is that in general ISH is considered positive if the ratio of the *HER2* gene copy number per tumor cell to the chromosome 17 copy number is greater than or equal to 2 (*HER2*/CEP17-Ratio ≥ 2) (Summary of Product Characteristics Herceptin^®^ i.v. 2015; Bang et al. 2010) based on a count of at least 20 cells. In borderline negative results just below the cut-off (ratio: 1.8 to <2.0) it is particularly advisable to evaluate an additional 20 tumor cells in the existing tumor specimen. If the finding is still borderline negative, a rebiopsy is recommended (see above). In surgical specimens, particularly those, which show intestinal histology, assessment of an additional alternative tumor block may also be considered.

## Comments on the availability (acquisition) of tumor blocks

From the participants’ perspective, the acquisition of samples/tumor blocks can be a challenge due to issues such as logistical co-ordination, the amount of time taken for the requested sample plus the initial findings to become available, and reimbursement. In principle, all clinicians are obliged to provide required documentation/findings to the clinician in charge of a patient’s follow-on treatment. This also includes histopathology samples (paraffin blocks). Accordingly, any pathologist is obliged to provide tumor blocks upon a clinician’s request where determining HER2 status is relevant to treatment decision-making. Some countries have specific payment rules for shipping (Empfehlungen zur Konsiliar- und Zweitbefundung in der Pathologie des Bundesverbandes Deutscher Pathologen und der Deutschen Gesellschaft für Pathologie 2010). Requested samples should be sent for testing as soon as possible, i.e. within 1–2 days. Consideration should be given to the fact that non-availability of samples leads to increased medical burden and costs, because the patient would be required to undergo another gastroscopy or biopsy.

## Specific considerations for HER2 testing and treatment decisions from the clinician’s perspective

### Consensus

HER2 testing should be requested by the clinician prior to deciding on first-line treatment for metastatic gastric cancer.

### Consensus

If the clinician does not actively request HER2-testing, pathologists should check with the clinician whether HER2 testing is required for a treatment decision when submitting their findings.

HER2-testing should be quality-assured. To support this, samples should be sent to pathology laboratories which are regularly and successfully participating in interlaboratory (proficiency or ‘round robin’) quality assurance schemes for HER2 testing of stomach cancer, such as that offered by the Quality-Assurance Initiative Pathology (QuIP^®^) in collaboration with the Reference Institute for Bioanalytics (RfB) in Germany (consensus).

- Various findings were discussed
- In case of a HER2-negative primary finding, a rebiopsy should be carried out if anti-HER2 targeted therapy may be indicated in case of relapse (i.e. where metastases have occurred) (consensus).
  - If the metastasis is accessible, rebiopsy should be performed provided this can be tolerated by the patient (consensus).
  - If the metastasis is not accessible for biopsy, any additional archived material (biopsy, surgical specimens) should also be accessed if available, and the primary finding should be confirmed by testing of this additional material (consensus).
- In case of a HER2-positive primary finding and a HER2-negative metastasis, the positive finding remains valid, and there is the option for treatment with trastuzumab.

## False positive and false negative HER2 testing: causes and solutions

The problem of tumor heterogeneity and dealing with discordant findings between biopsy and surgical specimens were discussed.

### Consensus

In the case of discordant findings between biopsy and surgical specimens, if one of the specimens is HER2-positive, then the tumor should be evaluated as positive.

Evaluation of the HER2 status can be performed on the surgical specimen and the endoscopic biopsy. The scoring system for the assessment of IHC staining pattern in gastric cancer includes both sample types, but gives different criteria for categorization of HER2 status between biopsy and surgical specimen (see Table 1). The results of the evaluation of HER2 overexpression in the surgical specimen may differ from the evaluation of the biopsy (discordant finding). The high intratumoral heterogeneity of gastric carcinoma with focal forms of HER2 expression, which is found in approximately 30% of HER2-positive cases, is a potential cause of discordant findings.

## Quality assurance in HER2 testing of gastric carcinoma

The trastuzumab Summary of Product Characteristics states that HER2 testing for the detection of HER2 overexpression or *HER2* gene amplification in gastric cancer must be performed in a specialized laboratory that can ensure adequate validation of testing procedures (Summary of Product Characteristics Herceptin^®^ i.v. 2015). To ensure validation of testing procedures and the generation of accurate and reproducible results the laboratory must be staffed by trained personnel (Summary of Product Characteristics Herceptin^®^ i.v. 2015).

Sufficient experience in the histopathological evaluation of gastric carcinoma is important because there are significant differences in the evaluation of HER2 test results between breast and gastric cancer. In particular, the immunohistochemical characteristics of gastric carcinoma differ due to high intratumoral heterogeneity and a gastric cancer-specific staining pattern. Simple transferal of criteria for the immunohistochemical HER2 evaluation from breast cancer to gastric cancer would result in a high false negative rate in gastric cancer (Barros-Silva et al. 2009). One of the major differences is that the circular and complete membranous staining required to confirm HER2-positivity in breast cancer is rare in this form in gastric cancer and is not a defining characteristic for HER2-positivity in this setting (Barros-Silva et al. 2009). Therefore, the evaluation criteria established prior to the ToGA study specifically for gastric cancer for valid HER2 testing must be strictly adhered to (Bang et al. 2010; Hofmann et al. 2008).

Quality assurance initiatives are available in several countries. In Germany, the Quality Assurance Initiative (QuiP^®^) of the German Society of Pathology and the Federal Association of German Pathologists is available as an established tool to address the basic requirements of HER2 testing and recommendations of the guidelines. QuiP^®^ offers pathology laboratories in Germany the opportunity to participate in interlaboratory comparisons as part of external quality assurance system for tumor diagnostics (Information about QuiP^®^ 2016). In addition to such interlaboratory comparisons, pathology laboratories may use the Her2-MONITOR of the Medical School Hannover as an internal quality assurance tool. This allows for the documentation of HER2-positivity rates and comparison with the mean of all institutes participating in the Her2-MONITOR scheme (“benchmarking”) (Choritz et al. 2011). The HER2 monitor’s mean HER2 positivity rate is 20.42 ± 8.88% (as of Feb 24th, 2016) on the basis of 24 evaluable institutes and 3056 documented cases.

The experts recommend that pathology facilities should regularly (at least every 2 years) participate in interlaboratory comparisons for HER2 testing of gastric cancer. Clinicians should ascertain which pathology facilities are appropriately certified via successful participation in interlaboratory comparison quality assurance for HER2 gastric cancer (the certified institutes are listed on the home page of the German Society of Pathology, see http://www.dgp-berlin.de.) and should preferably submit samples for the determination of HER2 to such facilities.

## Participants of the expert meeting on September 21st, 2015 in Frankfurt a.M.

The expert meeting was held in Frankfurt a.M. on September 21st, 2015 at the invitation of the Roche Pharma AG. The recommendations referred to in this manuscript are the consensus of all participants and reflect their opinions and long-term experience in this field.

*Clinicians* Lordick M, Al-Batran S, Hofheinz R, Lorenzen S, Möhler M, Thuss-Patience P.

*Pathologists* Baretton G.B., Dietel M, Gaiser T, Kreipe H.H., Kirchner T, Quaas A, Röcken C, Rüschoff J, Tannapfel A.
